# Case Report: Double lumen tube insertion in a morbidly obese patient through the non-channelled blade of the King Vision ^™^ videolaryngoscope

**DOI:** 10.12688/f1000research.4481.3

**Published:** 2014-08-08

**Authors:** Mohamed El-Tahan, D. John Doyle, Alaa M Khidr, Ahmed G Hassieb

**Affiliations:** 1 Department of Anesthesiology, King Fahd Hospital, Dammam University, Al Khubar, 31952, Saudi Arabia; 2 Department of General Anesthesiology, Cleveland Clinic, Cleveland, OH, 44195, USA

## Abstract

We describe the insertion of the double lumen endobronchial tube (DLT) using a non-channeled standard blade of the King Vision ^TM ^videolaryngoscope for one lung ventilation (OLV) in a morbidly obese patient with a predicted difficult airway, severe restrictive pulmonary function, asthma, and hypertension. The patient was scheduled for a video-assisted thoracoscopic lung biopsy. The stylet of the DLT was bent to fit the natural curve of the #3 non-channeled blade of the King Vision ^™^ videolaryngoscope. We conclude that the use of King Vision ^™^ videolaryngoscope could offer an effective method of DLT placement for OLV.

## Introduction

The GlideScope ^®^ (Verathon Inc., Bothell, WA, USA) has been used to facilitate the placement of the double lumen endobronchial tubes (DLT) in patients with a difficult airway ^1, 2^. However, DLT placement in patients with a limited mouth opening is relatively difficult compared to a single-lumen tube (SLT) because of the larger outer diameter, the distal curvature and the increased rigidity ^3, 4^. The DLT version of the channeled Airtraq ^®^ laryngoscopes (Prodol Limited, Viscaya, Spain) is equivalent in performance to direct laryngoscopy with a Macintosh blade ^4^.

The King Vision™ video laryngoscope (King Systems, Indianapolis, IN, USA) is a portable video laryngoscope (VL) similar to the Pentax Airway Scope ^®^ (Pentax-AWS, Hoya Corp., Tokyo, Japan), but different in that the LED light and CMOS camera are part of the disposable blades. These blades are available in two styles: a standard non-channeled blade that requires the use of a stylet shaped to 60–70° to direct the SLT, and a channeled blade that incorporates a guide channel which directs the SLT towards the glottis. Both designs include an anti-fog lens coating. The height and width of the standard non-channeled and channeled blades are 13 mm and 26 mm vs. 18 mm and 29 mm, respectively. Among the Airtraq ^®^, the Pentax Airway Scope ^®^ and the King Vision™ VL, the standard non-channeled blade of the King Vision™ VL has the smallest diameter.

In this report we show how the use of the standard non-channeled blade of the King Vision™ videolaryngoscope can be useful for DLT placement, as illustrated in the management of a morbidly obese patient with predicted difficult airway and severely restrictive pulmonary dysfunction.

## Case report

A 52 year-old, 151 cm, 95 kg (body mass index 41.7 kg/m ^2^) Asian woman presented with progressive orthopnea, dyspnea, and cough and was admitted to hospital. She had a 15 years history of moderate asthma, hypertension and hypocalcemia and was treated with irbesartan 150 mg/day, furosemide 40 mg/day, calcium carbonate 1.2 g/day and inhaled salbutamol.

On physical examination, dyspnea on mild exertion was present. The respiratory rate (RR) was 17/min, the resting heart rate (HR) was 80/min, blood pressure (BP) was 150/90 mmHg and arterial oxygen saturation (SpO _2_) was 90% on a room air. Examination of the other systems (including abdomen and central nervous system examinations) revealed no abnormalities. Preoperative airway examination revealed a Mallampati class III airway, with an intercisor distance of 3.5 cm, a thyromental distance of 6.0 cm, normal teeth, and a full range of neck flexion and extension.

Chest radiography showed reticular opacities with honeycombing. Electrocardiography showed left axis deviation and poor R wave progression. Transthoracic echocardiography showed impaired left ventricular relaxation, mild apical wall hypokinesis and an ejection fraction of 0.55. The patient’s electrolytes and creatinine were normal. Hemoglobin concentration was 12.9 g/dl and ionized calcium was 0.7 mmol/l. Pulmonary function testing showed a severe restrictive pattern (forced expiratory volume in first second [FEV _1_] 44.5%, forced vital capacity [FVC] 40.5%, and FEV _1_/FVC 109% of predicted). Arterial blood gases analysis showed hypoxemia on room air (pH 7.39, PaCO _2_ 46.6 mmHg, HCO _3_ 27.7 mmol/l, PaO _2_ 58 mmHg).

The patient was scheduled for a video-assisted thoracoscopic lung biopsy. Multidisciplinary discussions involving a cardiothoracic surgeon, a pulmonologist, anesthesiologists and the family of the patients took place, emphasizing the possibility of acute pulmonary compromise during tracheal intubation and surgery. Awake fibreoptic intubation was offered as the best airway management option, but the patient refused. Written informed consent was obtained for tracheal intubation after induction of general anesthesia with the adopted stepwise plan.

A stepwise plan was formulated: the initial plan included induction of general anesthesia through the placement of a left DLT using the King Vision™ VL. Backup plans were revised involving the insertion of the left DLT over a placed Eschmann tracheal tube introducer (Smiths-Medical International Ltd, Hythe, Kent, UK), and using a King Vision™ VL, an Arndt’s endobronchial blocker placed through a SLT. The use of selective lobar blockade was considered, if needed to correct hypoxemia during lung ventilation.

Oxygen at 3 L/min was delivered via a nasal cannula inserted upon entry of the patient in the operating room. Glycopyrrolate 0.2 mg was administered intravenously. Patient monitoring included electrocardiography, pulse oximetry, invasive arterial blood measurement, capnography, train of four stimulation of the ulnar nerve, and entropy-based depth of anesthesia monitoring. A left thoracic paravertebral catheter was inserted. No sedative premedication was given.

After positioning of the patient on the operating table in a head-up position, anesthesia was induced using a target-controlled infusion (TCI) of sufentanil with a target effect site concentration (Ce) of 0.1 ng/mL, in conjunction with 8% sevoflurane in 92% oxygen delivered by mask ventilation using pressure-support ventilation of 15 cm H _2_O and with an Aisys anesthesia care station (GE Healthcare, Helsinki, Finland). The patient was easily ventilated using bag mask ventilation (Han *et al.* grade 1) ^5^. This lasted about 8 min.

Laryngoscopy was performed using a King Vision™ videolaryngoscope, where a grade II view of the glottis was observed. Succinylcholine (80 mg) was then administered intravenously for muscle relaxation.

**Figure 1.**
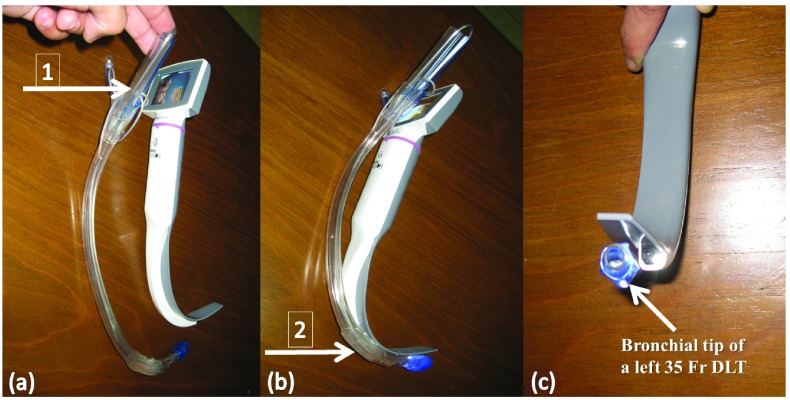
Photograph of a “standard” non-channeled blade of the King Vision™ videolaryngoscope with a stylet placed in a left 35 Fr double-lumen tube (DLT) to match the contour of the blade. ( **a**) Arrow (1) shows how the proximal DLT curve remains directed to the right side. ( **b**) Arrow (2) shows how the distal DLT curve follows the curve of the standard non-channeled blade (approximately 60–70°). ( **c**) Shows the bronchial tip of the DLT adapted to the tip of the standard non-channeled blade.

The stylet of a 35 Fr left DLT (Portex ^®^ Blueline Endobronchial tube, Smiths Medical Intl. Ltd., Hythe, Kent) was bent to fit the natural curve of a standard non-channeled blade of a King Vision™ VL [ Figure 1A–C]. After mask ventilation, a second laryngoscopy with the introduction of the standard blade of a King Vision™ VL through the mouth followed with gliding of the left DLT over the posterior surface of the standard non-channeled blade.

**Figure 2.**
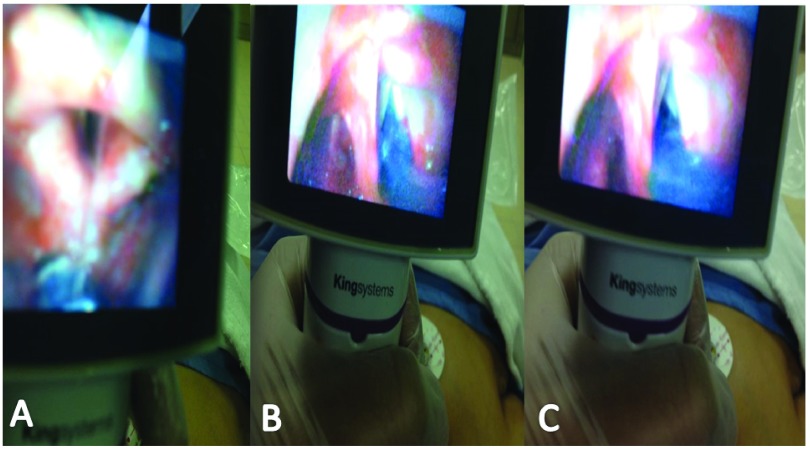
Photograph showing a bronchial tip of a left 35 Fr double-lumen tube (DLT) passing towards ( **A**) and through ( **B**) the vocal cords, and ( **C**) following removal of the stylet and 180° counterclockwise rotation of the DLT through the display unit of a King Vision™ videolaryngoscope.

After satisfactory visualization, the left DLT was directed through the glottic opening into the trachea [ Figure 2A–C]. The operator’s index finger prevented the perforation of the tracheal cuff of the DLT by the sharp upper teeth during passage through the mouth opening. The stylet was then removed and the DLT rotated counterclockwise 180° and advanced to the 27 cm mark at the incisors, while the glottis was visualized via the King Vision™ VL. The DLT position was verified fibreoptically.

The time from the cessation of mask ventilation until resumption of mechanical ventilation following intubation was about 90 s. The lowest SpO _2_ during intubation was 95%.

Anesthesia was maintained with sevoflurane (0.8–0.9 minimum alveolar concentration), TCI sufentanil with a Ce of 0.1 ng/mL and cisatracurium 5 mg. Transient severe hypotension (BP was 57–78/42–52 mm Hg that lasted for 25 min) was treated with reducing the sufentanil Ce to 0.05 ng/ml, and administering boluses of 6% hydroxyethyl starch 130/0.4 (Voluven ^®^ 6%, Hospira, Fresenius Kabi, Halden, Norway), as well as phenylephrine (300 µg) and ephedrine (10 mg).

The patient’s right lung was ventilated in pressure-controlled ventilation mode, with FiO _2_ set at 0.7, a delivered tidal volume (TV) of 360 mL, an inspiratory-to-expiratory [I: E] ratio of 1:2, PEEP of 5 cm H _2_O, and RR of 14–16/min. The peak airway pressure (Ppk) was limited to 35 cm H _2_O and a fresh gas flow (FGF) of 1.6 L/min was used. Neither continuous positive airway pressure (CPAP) nor high frequency positive pressure ventilation (HFPPV) was needed for the non-dependent lung ^6^; SpO _2_ was maintained over 92% during 25 minutes of one lung ventilation (OLV). The operation proceeded uneventfully, with excellent lung isolation.

After the surgery, the residual effects of neuromuscular blockade were reversed with neostigmine 2.5 mg and glycopyrrolate 0.6 mg. The patient was extubated and post-operative analgesia was accomplished with a continuous infusion of bupivacaine 0.125% through the paravertebral catheter. A post-operative follow-up (for the next six days after surgery) showed no evidence of hoarseness.

## Discussion

Two main techniques can be used to achieve lung isolation in patients with a predicted difficult airway: [1] using a DLT or [2] using a bronchial blocker inserted through a SLT. There is no overall advantage of either over the other in the morbidly obese patient ^7^.

Our patient had predictable hypoxemia during OLV because of a severe restrictive pulmonary dysfunction and a low PaO _2_; despite this, significant hypoxemia was not noted during the relatively short period of OLV ^8^. A DLT was chosen over a bronchial blocker so that the non-ventilated non-dependent lung could be oxygenated using HFPPV, although a bronchial blocker could have been used to provide CPAP to the non-ventilated non-dependent lung ^6^. Additionally, a DLT allows effective bilateral suctioning. The difficulty in surgical access precluded the use of selective lower lobar collapse, which could have been helpful to correct the predicted intraoperative hypoxemia during OLV. In a manner similar to others ^9, 10^, pressure support pre-oxygenation was used to improve baseline arterial oxygen saturation before and during induction of general anesthesia; this succeeded in maintaining SpO _2_≥95% during intubation.

The true difficulty of intubation using standard direct laryngoscopy remains unknown. We chose not to attempt direct laryngoscopy prior to the use of the King Vision™ VL. First, the patient had three predictors of an anticipated difficult airway, including a BMI of 41.7 kg/m ^2^, a short thyromental distance and a limited mouth opening. Second, we were concerned about the possibility of hypoxemia in case of extended time of laryngoscopy and intubation due to a prior assessment of the airway using direct laryngoscopy. We thus felt that prior assessment of the airway using direct laryngoscopy would have not changed the adopted plan and would have conferred little benefit. Third, the recent ASA Guidelines for Management of the Difficult Airway ^11^ do not suggest a prior assessment using direct laryngoscopy. The guidelines also suggest the use of VL as a choice for tracheal intubation in the non-emergent pathway where ventilation is adequate. Finally, an improved view of the glottis does not always translate into easier tracheal intubation. The ease of intubation depends on many factors: (i) the type and size of the videolaryngoscope; (ii) the experience of the laryngoscopist; (iii) the use of external laryngeal manipulation; (iv) the optimization of head positioning; and (v) the use of adjuncts such as a stylet ^12^. In the present case, endobronchial intubation was successfully performed during the first intubation attempt by an anesthesiologist who has an experience with more than 200 tracheal intubations using King Vision™ VL.

Videolaryngoscopy can sometimes facilitate DLT insertion compared with direct laryngoscopy ^13, 14^. Other investigators have reported 17% to 27% reductions in time to intubation with a DLT compared with direct laryngoscopy ^15, 16^. Wojtczak recommended the routine use of videolaryngoscopy during all elective intubations to avoid any trauma from failed direct laryngoscopy, as well as to reduce the patient’s stress response to intubation (and our own stress) ^17^. Channeled VLs have a number of advantages over those with angulated blades, such as the GlideScope ^®^, and has been found to shorten the time taken to position the endotracheal tube in a small manikin study ^18^. Channeled VLs have a passage to guide the SLT; thus, once an adequate view of the glottis has been obtained, the VL is kept steady and the SLT advanced into the glottis with the right hand. By contrast, the angulated blade design uses a different technique for placing the SLT: once an adequate view of the glottis is obtained, the operator holds the laryngoscope with the left hand and manipulates the SLT into the glottis with the right hand using the view on the screen is used as a guide ^18^.

Channeled videolaryngoscopes are more suitable in patients with a limited mouth opening compared to traditional videolaryngoscopes like the GlideScope ^®^^19– 21^.

The King Vision™ VL accommodates a minimum mouth opening of 13 mm for the standard non-channeled blade and 18 mm for the channeled blade. Studies in manikins point to improved laryngeal visualization with the use of the Airtraq ^®^ VL over the GlideScope ^®^^22– 24^.

However, the large outer diameter and more rigid design of DLTs make them relatively harder to insert it through classic channeled blades. This requires either the use of a specific videolaryngoscope design like the DLT version of the Airtraq ^®^^4^, or the use of a tube exchanger over which a large DLT can be placed ^25^. The DLT Airtraq ^®^ laryngoscope is available for the 35 Fr to 41 Fr DLTs. However, it has not gained widespread popularity because it requires a minimum mouth opening of 19 mm, provides only subtle enhancement of visualization, has a higher incidence of hoarseness over the Macintosh laryngoscopes ^4^, and has a narrower field of view than King Vision™ VL (80° vs. 160°) ^26^. Regardless, a superior field of view does not necessarily result in an improved view of the laryngeal inlet, or leads to easier insertion of the tracheal tube ^4^.

Suzuki *et al.* described the removal of the tube channel back plate of the Airway Scope ^®^ for intubation with a 39 Fr DLT in a patient with unpredicted difficult intubation and inadequate mouth opening ^27^. Compared with the Airtraq ^®^ and the Pentax Airway Scope ^®^, the standard non-channeled blade King Vision™ VL has the thinnest and shortest stature (26 mm vs. 28 mm and 49 mm and 13 mm vs. 18 mm and 131 mm, respectively) and the widest field of view (160° vs. 80° and 90°, respectively), the dimensions of which may make it superior for those with limited mouth opening ^26– 28^.

Although the use of video laryngoscopy for placement of DLTs has been well described, the present report describes a novel approach to DLT intubation and offers another tool for patients who require lung isolation. The standard non-channeled blade of the King Vision™ VL could provide a new mean for insertion of DLTs in patients with a minimum mouth opening of 13 mm. This approach offers a 160° field of view, potentially facilitating the manipulation and rotation of the DLT upon visualization.

Akihisa *et al.* ^29^ have reported longer intubation times with the use of the non-channeled King Vision™ laryngoscope compared to the conventional Macintosh laryngoscope or the channeled King Vision™ video laryngoscopes. However, the results of that particular study cannot readily be applied to the present case report because the operators were non-experienced nurses who had never previously performed a tracheal intubation (rather than anesthesiologists with over 10 years of experience, as in the present report). In addition, the Akihisa study was done on manikins with simulated normal airways rather than patients with a difficult airway, and it tested the efficacy of the tested devices using a single lumen tube rather than a larger double lumen tube.

Here we described the necessary maneuvers to insert a DLT using a standard non-channeled blade of King Vision™ VL. We recommend four steps: first, bend the DLT stylet so that the distal 21 cm of the DLT curve follows the curve of the standard non-channeled blade and the proximal curve of the DLT remains directed to the right side. Next, insert the DLT, exercising caution to avoid damage to the tracheal cuff by the upper teeth during its passage through the mouth opening. Then, after the bronchial cuff passes through the vocal cords, withdraw the stylet of the DLT. Finally, rotate the DLT 180° counterclockwise while advancing the DLT to the desired depth. In conclusion, the use of King Vision™ videolaryngoscope could offer an effective method of DLT placement for OLV.

## Consent

The patient provided informed written consent for the publication of this report.
