# Supplementary material for: Case Report: Double lumen tube insertion in a morbidly obese patient through the non-channelled blade of the King Vision ™ videolaryngoscope
Source: F1000Res. 2014 Aug 8;3:129. Originally published 2014 Jun 17. [Version 3] doi: 10.12688/f1000research.4481.3 (PMC4184295; doi:10.12688/f1000research.4481.3)
Supplement: Peer review history

# Peer review: Referee response for version 3

**DOI:** 10.5256/f1000research.5242.r5755

**Published:** 2014-09-25

**Authors:** David Healy

**Affiliations:** 1 Department of Anesthesiology, University of Michigan, Ann Arbor, MI, USA

Thank you for giving me the chance to review this well-written and interesting case report with comment.

The authors have made significant efforts to clarify the case, and modify their introduction and discussion points.

I approve this manuscript, but I have 2 remaining reservations which I would like the authors to consider:

The discussion retains two statements that should be qualified with the word "may" as the evidence for the statements are limited, namely:

1.  "Channeled VLs have a number of advantages over those with angulated blades, such as the GlideScope®" - They may have advantages, but the evidence remains weak, and is likely dependent (as previously stated by the authors, to device familiarity and availability).
2. "Channeled videolaryngoscopes are more suitable in patients with a limited mouth opening compared to traditional videolaryngoscopes like the GlideScope®" - Again, they may have advantages, but the evidence remains weak. Because a device can "fit through the incisor gap" doesn't necessarily mean it performs better when placing a tube into the trachea.

Again, I do not wish to hold up the indexing of this interesting case study based on pedantics but I feel we must be careful when providing broad comments that may be falsely reassuring, and lead to incorrect assumptions of the performance of one device of another.

I have read this submission. I believe that I have an appropriate level of expertise to confirm that it is of an acceptable scientific standard.

# Peer review: Referee response for version 2

**DOI:** 10.5256/f1000research.5142.r5496

**Published:** 2014-07-22

**Authors:** David Healy

**Affiliations:** 1 Department of Anesthesiology, University of Michigan, Ann Arbor, MI, USA

Thank you for the opportunity to re-review this manuscript following the authors’ changes, additions, and clarifications. As before, this is a well written discussion of a challenging airway management scenario. The manuscript is greatly improved with the authors careful edits. I do however, have several reservations with the manuscript primarily related to unsupported statements and low quality references.

**Technical issues:**

I appreciate the authors clarification regarding the previous technical concerns. Thank you for drawing my attention to the ability to bag mask ventilate expressed as “mask ventilation using pressure-support ventilation of 15cm H _2_O” - to clarify, does the manuscript mean manual airway support, the application of a face mask, and the ventilator turned on?

The degree of difficulty in ability to bag mask ventilate changes the entire clinical context of this patient’s airway management. I suggest this is expressed according to the Han grade for clarification ( Hans *et al*., 2004).

I suggest the inclusion of a statement regarding the fact that the true difficulty of intubation (according to the standard of direct laryngoscopy) remains unknown. The authors have explained the reasons for progressing directly to videolaryngoscopy, but being at high risk of difficulty is distinct from true difficulty. In response to the authors reply previous reply, the ASA guidelines do not actually recommend the use of videolaryngoscopy in a predicted difficult intubation - they suggest the consideration of it. This is an important, but admittedly subtle, distinction. I agree the guidelines do not specifically recommend or suggest (see previous) a check DL view before progression to videolaryngoscopy, but it does not make it a bad idea (especially in a stable patient receiving adequate bag mask ventilation).

**Content issues:**

Please consider removal of the statement “Channelled video laryngoscopes are more suitable in patients with a limited mouth opening compare to traditional videolarygoscopes like the GlideScope”. This may or may not be true, but it isn’t supported by the current reference, which describes a manikin study. e.g. to be correct would need to rephrase “Channelled video laryngoscopes have been shown to be advantageous when performing intubations in manikins with limited mouth opening…” which is more accurate, but reduces the relevance of the statement.

Please consider removal of the statement “the King Vision view was a grade II, therefore the CL view would be higher”. This is speculative. As stated previously the DL C&L view remains unknown as it was never performed, and can not be estimated from the visualization findings of a different device.

Please consider removal or rephrase the statement “Channeled VLs have a number of advantages over those with angulated blades, such as the GlideScope, and may shorten the time taken to position the endotracheal tube” - this is referenced with a manikin study, to be correct this should be rephrased “and was found to shorten the time taken to position the endotracheal tube in a small manikin study”

Please consider clarification of the referenced statement, “Previous studies have demonstrated the Airtraq VL to provide improved laryngeal visualization of than the GlideScope.” The manuscript refers to 2 manikin studies, and one prospective study where no statistically significant difference in grade I/II vs III/IV between the GlideScope and Airtraq groups were found. For inclusion this should be phrased “Studies in manikins have suggested an improved laryngeal visualization with use of the Airtraq over the GlideScope”. Again this is more accurate, but reduces the relevance of the statement.

Please consider modifying the statement “that makes it superior for those with limited mouth opening” to “the dimensions of which may make it superior…” as the referenced articles do not address this statement.

I have read this submission. I believe that I have an appropriate level of expertise to confirm that it is of an acceptable scientific standard, however I have significant reservations, as outlined above.

# Response

**Published:** 2014-07-26

**Authors:** Mohamed El Tahan

**Affiliations:** Dammam University, Saudi Arabia

We read with interest the important comments of Dr. David Healy, Department of Anesthesiology, University of Michigan, Ann Arbor, MI, USA. Here are our responses to his comments:

**Technical Issues:**

1. **Dr. Healy: **"Thank you for the opportunity to re-review this manuscript following the authors’ changes, additions, and clarifications. As before, this is a well written discussion of a challenging airway management scenario. The manuscript is greatly improved with the authors careful edits. I do however, have several reservations with the manuscript primarily related to unsupported statements and low quality references."
**Authors':** Thank you so much. We addressed your reservations one by one as follows:
2. **Dr. Healy: **"I appreciate the authors clarification regarding the previous technical concerns. Thank you for drawing my attention to the ability to bag mask ventilate expressed as “mask ventilation using pressure-support ventilation of 15cm H _2_O” - to clarify, does the manuscript mean manual airway support, the application of a face mask, and the ventilator turned on? "
**Authors:** That is correct. We meant that a face mask was applied and a pressure-support ventilation mode of an Aisys anesthesia care station (GE Healthcare, Helsinki, Finland).
3. **Dr. Healy: **"The degree of difficulty in ability to bag mask ventilate changes the entire clinical context of this patient’s airway management. I suggest this is expressed according to the Han grade for clarification ( Hans et al., 2004).”
**Authors:** The present case had only one predictor of difficult mask ventilation, that was the body mass index of 41.7 kg/m ^2^.[ref-1] The patient was easily ventilated by a bag mask ventilation (Hans grade 1). Similar to others[ref-2], a pressure support preoxygenation was used in the present case to improve her baseline low arterial oxygen saturation before and during induction of general anesthesia, that succeeded to maintain SpO _2_ ≥95% during intubation.
4. **Dr. Healy: **"I suggest the inclusion of a statement regarding the fact that the true difficulty of intubation (according to the standard of direct laryngoscopy) remains unknown."
**Authors:** Well, we added a statement as follows “The true difficulty of intubtion according to the standard of direct laryngoscopy remains unknown”. However, we have few comments about that raised point. First;  Cavus *et al. *[ref-3], have reported improved Cormack and Lehane laryngoscopy grades from 3 or 4 to 1 or 2a with the use of C-Mac VL D blade. Thus,  the use of VLs is quite unlike intubation via direct vision with a standard Macintosh blade where the challenging part of the process is usually obtaining the appropriate Cormack and Lehane view, rather than passing the endotracheal tube into the trachea *per se*.[ref-4] Second, the improved view could not always be translated into easier tracheal intubation. The ease of intubation depends on many factors like as (i) name and size of the videolaryngoscope; (ii) grade/experience of the laryngoscopist; (iii) number of attempts; (iv) optimising position/use of adjuncts; and (v) easy/difficult/failed intubation.[ref-5] In the present case, endobronchial intubation was successfully performed during the first attempt of intubation by an anesthesiologist who has an experience with more than 200 tracheal intubations using King Vision VL. Third, the limited mouth opening in the present case would preclude the easy passage of  a 35 Fr left DLT with its larger outer diameter than a 8.0 mm SLT.
5. **Dr. Healy: **"The authors have explained the reasons for progressing directly to videolaryngoscopy, but being at high risk of difficulty is distinct from true difficulty.
**A** **uthors:** That is correct. Please, refer to the 2 ^nd^ point in our response (no. 4). Additionally, in the view of the low baseline values of SpO _2 _in the present case, we planned to proceed directly with King Vision to shorten the duration of laryngoscopy and endobronchial intubation and hence the risk of worsening of her noted hypoxemia. Similarly, other investigators have reported 17% to 27% shorter time to intubation with a DLT compared with the direct laryngoscopy in elective thoracic surgery.[ref-6] ^-^[ref-7] ****Wojtczak recommended a routine use of videolaryngoscopy during all elective intubations to avoid trauma of failed direct laryngoscopy, reduce the patient’s stress response to intubation and our own stress, and to avoid losing valuable seconds to retrieve a unit.[ref-8]
6. **Dr. Healy: **"In response to the authors reply previous reply, the ASA guidelines do not actually recommend the use of videolaryngoscopy in a predicted difficult intubation - they suggest the consideration of it. This is an important, but admittedly subtle, distinction."
**Authors:** Fortunately, the An Updated Report by the American Society of Anesthesiologists Task Force on Management of the Difficult Airway included “the use of video-assisted laryngoscopy as an initial approach to intubation”, in patients with predicted difficult airway after induction of anesthesia in the Non-Emergency Pathway (Appendix 1: Summary of Recommendations, Page 259, Column 2, Lines 11-12)
7. **Dr. Healy: **"I agree the guidelines do not specifically recommend or suggest (see previous) a check DL view before progression to videolaryngoscopy, but it does not make it a bad idea (especially in a stable patient receiving adequate bag mask ventilation).”
**Authors:** We agree that it could be not a good idea. However, it does not meet the standards of care for such cases, especially after the revolution of VLs.

**Content Issues:**

1. **Dr. Healy: **“Please consider removal of the statement “Channelled video laryngoscopes are more suitable in patients with a limited mouth opening compare to traditional videolarygoscopes like the GlideScope”. This may or may not be true, but it isn’t supported by the current reference, which describes a manikin study. e.g. to be correct would need to rephrase “Channelled video laryngoscopes have been shown to be advantageous when performing intubations in manikins with limited mouth opening…” which is more accurate, but reduces the relevance of the statement.”
**Authors:** We amended it. We changed the references. Other investigators have reported difficulty in introducing of the SLT in patients with a limited mouth opening, despite the obtained clear view of the glottis with the non-channelled Glidescope due to the wide and square design of its blade.[ref-9] The limitation in mouth opening could has the potential to break the disposable plastic GVL ^®^ Stat blade of a Glidescope during repositioning of the handle back to midline.[ref-10] However, a limited mouth opening in a morbidly obese patient with a BMI > 40 kg/m ^2^ might be responsible for broken of the disposable plastic GVL ^®^ Stat blade of a non-channelled Glidescope during repositioning of the handle back to midline.[ref-11]
2. **Dr. Healy: **“Please consider removal of the statement “the King Vision view was a grade II, therefore the CL view would be higher”. This is speculative. As stated previously the DL C&L view remains unknown as it was never performed, and can not be estimated from the visualization findings of a different device.”
**Authors:** We removed it.
3. **Dr. Healy: **“Please consider removal or rephrase the statement “Channeled VLs have a number of advantages over those with angulated blades, such as the GlideScope, and may shorten the time taken to position the endotracheal tube” - this is referenced with a manikin study, to be correct this should be rephrased “and was found to shorten the time taken to position the endotracheal tube in a small manikin study”
**Authors:** We amended it.
4. **Dr. Healy: **“Please consider clarification of the referenced statement, “Previous studies have demonstrated the Airtraq VL to provide improved laryngeal visualization of than the GlideScope.” The manuscript refers to 2 manikin studies, and one prospective study where no statistically significant difference in grade I/II vs III/IV between the GlideScope and Airtraq groups were found. For inclusion this should be phrased “Studies in manikins have suggested an improved laryngeal visualization with use of the Airtraq over the GlideScope”. Again this is more accurate, but reduces the relevance of the statement.”
**Authors:** We amended it.
5. **Dr. Healy: **“Please consider modifying the statement “that makes it superior for those with limited mouth opening” to “the dimensions of which may make it superior…” as the referenced articles do not address this statement.”
**Authors:** We amended it.

[References]

[[1|type=journal|title=Prediction of difficult mask ventilation|authors=Langeron/O;Masso/E;Huraux/C;Guggiari/M;Bianchi/A;Coriat/P;Riou/B|source=Anesthesiology|year=2000|vol=92|issue=5|fpage=1229|lpage=1236|pmid=10781266|url=http://journals.lww.com/anesthesiology/pages/articleviewer.aspx?year=2000&issue=05000&article=00009&type=abstract]]

[[2|type=journal|title=Continuous positive airway pressure/pressure support pre-oxygenation of morbidly obese patients.|authors=Harbut/P;Gozdzik/W;Stjernfalt/E;Marsk/R;Hesselvik/JF|source=Acta Anaesthesoil Scand|year=2014|vol=58|issue=6|fpage=675|lpage=680|pmid=24738713 |doi=10.1111/aas.12317|url=http://onlinelibrary.wiley.com/doi/10.1111/aas.12317/abstract;jsessionid=FEF9DF4140DAEC29FB589BE80E5A1895.f04t01]]

[[3|type=journal|title=First clinical evaluation of the C-MAC D-Blade videolaryngoscope during routine and difficult intubation.|authors=Cavus/E;Neumann/T;Doerges/V;Moeller/T;Scharf/E;Wagner/K;Bein/B;Serocki/G|source=Anesth Analg|year=2011|vol=112|issue=2|fpage=382|lpage=385|pmid=21156978 |doi=10.1213/ANE.0b013e31820553fb|url=http://journals.lww.com/anesthesia-analgesia/pages/articleviewer.aspx?year=2011&issue=02000&article=00017&type=abstract]]

[[4|type=journal|title=Use of Cormack and Lehane grading with videolaryngoscopy|authors=Gray/H|source=Anaesth Intensive Care|year=2013|vol=41|issue=1|fpage=123|lpage=124|pmid=23362904 ]]

[[5|type=journal|title=Videolaryngoscopy and Cormack and Lehane grading.|authors=Angadi/SP;Frerk/C|source=Anaesthesia|year=2011|vol=66|issue=7|fpage=628|lpage=629|pmid=21682715 |doi= 10.1111/j.1365-2044.2011.06777.x|url=http://onlinelibrary.wiley.com/doi/10.1111/j.1365-2044.2011.06777.x/abstract]]

[[6|type=journal|title=Faster double-lumen tube intubation with the videolaryngoscope than with a standard laryngoscope|authors=Bensghir/M;Alaoui/H;Azendour/H;Drissi/M;Elwali/A;Meziane/M;Lalaoui/JS;Akhaddar/A;Kamili/ND|source=Can J Anaesth|year=2010|vol=57|issue=11|fpage=980|lpage=984|pmid=20857256 |doi=10.1007/s12630-010-9372-7|url=http://link.springer.com/article/10.1007%2Fs12630-010-9372-7]]

[[7|type=journal|title=Comparison of the GlideScope® videolaryngoscope and the Macintosh laryngoscope for double-lumen tube intubation.|authors=Hsu/HT;Chou/SH;Wu/PJ;Tseng/KY;Kuo/YW;Chou/CY;Cheng/KI|source=Anaesthesia|year=2012|vol=67|issue=4|fpage=411|lpage=415|pmid=22324297 |doi=10.1111/j.1365-2044.2011.07049.x.|url=http://onlinelibrary.wiley.com/doi/10.1111/j.1365-2044.2011.07049.x/abstract]]

[[8|type=journal|title=Should videolaryngoscopy be used routinely during all elective intubations?|authors=Wojtczak/JA|source=Minerva Anestesiol|year=2012|vol=78|issue=9|fpage=1076|lpage=1077|pmid=22337157|url=http://www.minervamedica.it/en/journals/minerva-anestesiologica/article.php?cod=R02Y2012N09A1076]]

[[9|type=journal|title=The modified ventilating tube changer to facilitate tracheal intubation using the GlideScope in patients with a limited mouth opening.|authors=Xue/FS;Yang/QY;He/N;Xu/YC|source=Br J Anaesth|year=2008|vol=101|issue=1|fpage=126|lpage=127|pmid=18556699|doi=10.1093/bja/aen155|url=http://bja.oxfordjournals.org/cgi/pmidlookup?view=long&pmid=18556699]]

[[10|type=journal|title=The clinical assessment of Glidescope in orotracheal intubation under general anesthesia.|authors=Xue/FS;Zhang/GH;Liu/J;Li/XY;Yang/QY;Xu/YC;Li/CW|source=Minerva Anestesiol|year=2007|vol=73|issue=9|fpage=451|lpage=457|pmid=17660737|url=http://www.minervamedica.it/en/journals/minerva-anestesiologica/article.php?cod=R02Y2007N09A0451]]

[[11|type=journal|title=Fracture of a GlideScope® Cobalt GVL® Stat disposable blade during an emergency intubation.|authors=Panacek/EA;Laurin/EG;Bair/AE|source=J Emerg Med|year=2012|vol=42|issue=6|fpage=125|lpage=128|pmid=19703743 |doi=10.1016/j.jemermed.2009.05.034|url=http://www.jem-journal.com/article/S0736-4679(09)00442-9/abstract]]

# Peer review: Referee response for version 2

**DOI:** 10.5256/f1000research.5142.r5495

**Published:** 2014-07-21

**Authors:** Maruyama Koichi

**Affiliations:** 1 Department of Anesthesiology, Teikyo University, Kawasaki, Japan

Thank you very much for your new version. In the revised version, I can confirm the author's great job for presenting detailed information about the patient's safety.

I have read this submission. I believe that I have an appropriate level of expertise to confirm that it is of an acceptable scientific standard.

# Peer review: Referee response for version 1

**DOI:** 10.5256/f1000research.4793.r5161

**Published:** 2014-07-07

**Authors:** David Healy

**Affiliations:** 1 Department of Anesthesiology, University of Michigan, Ann Arbor, MI, USA

Thank you for the opportunity to review this manuscript. The manuscript describes a single case report of the use of a video laryngoscope (King Vision non-channeled blade) to facilitate successful endobronchial intubation with a double lumen tube in an obese patients with respiratory comorbidity. The manuscript then discusses the characteristics of this particular videolayngoscope which the authors feel may lead to an improvement in success.

This case report is well written and adequately describes this challenging clinical scenario and airway management.

**Technical issues:**

1. There is no documentation of attempt to perform bag-mask ventilation during the case. There is no discussion of why this was not attempted or of its importance in the overall airway management of this patient. My view is the ability to perform adequate bag mask ventilation may improve the patient’s oxygenation and safety during the intubation attempts; especially it they were less straightforward than that described in this manuscript.
2. There was no attempt at direct laryngoscopy recorded. The actual difficulty of this patient’s airway remains unknown (e.g. she may have been easily ventilated with a bag mask and a grade I view at laryngoscopy with good positioning). She remains “at higher risk of difficulty” but the reality remains unknown.

 

**Content issues:**

1. I think the manuscript would benefit from a description of the other option of intubating a patient with a true difficult airway and significant pulmonary compromise: i.e. place a single lumen tube to improve oxygenation and ventilation, then exchange over an airway exchange catheter (Cook).
2. I think the comment on fluid choice is unnecessary.
3. Consider removing the phrase “Channeled VLs have many advantages over those with angulated blades, such as the GlideScope” - not true, and not referenced as so. Perhaps better described as “different advantages and limitations”
4. Consider removing the phrase “Previous studies…..more effective for DLT” - the references do not back up the link between an improved view and “potential successful intubation”. This comment can falsely reinforce the idea that view (on VL) equates to success.
5. I appreciate their relatively conservative summary statement at the articles conclusion - and the series of steps to achieve successful intubation.

I have read this submission. I believe that I have an appropriate level of expertise to confirm that it is of an acceptable scientific standard, however I have significant reservations, as outlined above.

# Peer review: Referee response for version 1

**DOI:** 10.5256/f1000research.4793.r5164

**Published:** 2014-06-30

**Authors:** Maruyama Koichi

**Affiliations:** 1 Department of Anesthesiology, Teikyo University, Kawasaki, Japan

Thank you for your case report demonstrating the effectiveness of non-channeled King Vision for DLT intubation in a morbidly obese patient with predicted difficult airway and severely restrictive pulmonary dysfunction.

I have a few comments and questions:

- Was the patient pre-oxygenated enough?  Oxygen at 3L/min via a nasal cannula could provide an approximate FiO _2_ of only 0.33. How long did it take from the cessation of mask ventilation until the resumption of the mechanical ventilation after intubation? In addition, could you tell me the lowest SpO _2_ during intubation?
- As you mentioned, the patient was easily expected to be exposed to the risk of severe hypoxia due to the morbid obesity and the comorbid pulmonary disease, as well as the predicted difficult airway. Furthermore, the time elapsed for intubation with the non-channeled King Vision would be longer than that with the conventional laryngoscopy or that with the channeled King Vision (Akihisa *et al.*, 2014).

The effectiveness of the non-channeled King Vision for DLT intubation was well described in this report. To clear the ethical issue, however, please provide the detailed information about the patient's safety.

I have read this submission. I believe that I have an appropriate level of expertise to confirm that it is of an acceptable scientific standard, however I have significant reservations, as outlined above.
